# Level of completion of a continuum of age-appropriate infant feeding practices and barriers among breastfeeding mothers in Ethiopia: a mixed methods study

**DOI:** 10.1186/s12889-024-17820-7

**Published:** 2024-02-01

**Authors:** Shikur Mohammed, Alemayehu Worku, Eshetu Girma

**Affiliations:** 1 School of Public Health, College of Health Sciences, Addis Ababa University, Addis Ababa, Ethiopia; 2 School of Public Health, St. Paul’s Hospital Millennium Medical College, Addis Ababa, Ethiopia

**Keywords:** Age-appropriate infant feeding, Exclusive breastfeeding, Complementary feeding, Ethiopia

## Abstract

**Background:**

Ethiopia has committed to ending undernutrition by implementing nutrition intervention strategies, including promoting optimal feeding and care practices. To monitor and evaluate optimal infant feeding practices, it is crucial to have reliable and quality data on infant feeding indicators. Therefore, this study aimed to evaluate the extent to which breastfeeding mothers in Ethiopia have completed the continuum of age-appropriate infant feeding practices and the barriers they face.

**Methods:**

In this study, a sequential explanatory mixed method design was used. First, using datasets from performance monitoring for action (PMA) in Ethiopia, we estimated the level of the outcome and associated factors. In the quantitative (QUAN) analysis, 1755 mothers of infants were included to generate estimates. A generalized estimating equations logistic regression model was used to identify factors associated with the outcome by accounting for the clustering nature of the data by enumeration area. Then, a qualitative (QUAL) study was conducted with 14 mothers to explore their infant feeding practices using an in-depth interview guide and analyzed using a thematic approach. Results from both quantitative and qualitative data were integrated, described under the identified thematic areas, and interpreted concurrently.

**Results:**

This study showed that 13.96% (95% CI: 12.4 to 15.6%) of mothers practiced a complete continuum of age-appropriate infant feeding. Over 8% of mothers did not practice any optimal feeding. Nearly 47% of mothers practiced optimal breastfeeding, and one-fifth of mothers practiced optimal complementary feeding. Results from both quantitative and qualitative data showed that mothers’ complete continuum of age-appropriate infant feeding practice was affected by their level of income, knowledge, and attitude towards optimal infant feeding, as well as by important others, including husbands, grandmothers, and health workers.

**Conclusion:**

The level of a complete continuum of age-appropriate infant feeding practice is low among breastfeeding mothers in Ethiopia. Mothers’ optimal feeding practices in Ethiopia are affected by their level of knowledge and attitude towards infant feeding, income or access to food, and health workers or family members. Therefore, collaborative efforts are needed to strengthen mothers’ education on the health benefits of optimal infant feeding and design and promote strategies to improve household income or access to diverse food.

## Background

Over one-third of infants worldwide have not been exclusively breastfed during the first four months of life [1, 2]. The rate of continued breastfeeding globally decreased from 74% in the first year of life to 46% in the second year of life. The recommended diverse diet was not given to over two-thirds of children aged 6 to 23 months. Feeding behaviors are suboptimal even in the richest households due to several reasons, including cultural factors and poor knowledge about optimal child feeding [2]. Only 59% and 14% of children in Ethiopia were exclusively breastfed and received adequate dietary diversity in 2019, respectively. In 2019, the proportion of infants exclusively breastfeeding decreased with age, from 73% in 0–1 months to 40% in 4–5 months [3–5].

Infant feeding practices that are inappropriate can be characterized by introducing complementary foods too early or too late, infrequent feeding, poor hygiene and feeding methods, and poor childcare practices [1]. Evidence has shown that the early introduction of complementary foods is associated with harmful effects, such as food allergies, the possibility of choking, a decrease in breast milk intake, childhood illness, and stunting [6–8]. The delayed introduction of complementary foods can cause anemia, food acceptance problems, and difficulty learning to eat [6–12]. According to experts, the potential health benefits of introducing complementary foods at six months of age outweigh any potential risk [6, 8, 12].

Evidence has shown that inappropriate infant feeding has significantly contributed to infant undernutrition, impaired development, and deaths in Sub-Saharan African countries, including Ethiopia [1, 13]. As a result of this, Ethiopia has committed to ending undernutrition by introducing and implementing nutrition intervention strategies, such as promoting optimal feeding in line with international recommendations [3, 14].

Optimal feeding practices are crucial to promoting healthy growth in infants and preventing childhood illnesses and undernutrition. Human breast milk can meet the nutrition needs of full-term and normal-birth-weight infants during the first six months of life. However, complementary foods should be introduced at six months of infant age because of the nutritional requirements of infants increase with age [1, 6, 9]. Timely and adequate complementary feeding in terms of amount, frequency, and diversity is recommended for children aged 6–23 months [6, 15, 16].

Previous research has demonstrated that mothers who gave birth at a health facility, had no economic problems and received breastfeeding counseling during pregnancy had a higher rate of exclusive breastfeeding. Moreover, mothers with higher educational levels and postnatal checkups were more likely to initiate complementary food properly [17–21]. However, the complete continuum of age-appropriate infant feeding practices and associated factors in Ethiopia is not well documented. Therefore, this study aimed to evaluate the extent to which breastfeeding mothers in Ethiopia have completed the continuum of age-appropriate infant feeding practices and the barriers they face.

## Methods

### Study settings

The quantitative study was conducted using the performance monitoring for action Ethiopia (PMA-Ethiopia) longitudinal panel survey datasets. PMA-Ethiopia survey was conducted in six regions that collectively represent 90% of the population in Ethiopia, namely, Addis Ababa, Oromia, Amhara, Tigray, the former Southern Nation Nationality People (SNNP) and Afar regions. The details of PMA-Ethiopia project design and settings can be accessed from the published PMA-Ethiopia project protocol [22].

The qualitative study was conducted in selected kebeles (the smallest administration unit in Ethiopia) in the health and demographic site (HDS) of Addis Ababa University and the rural community attachment site in two regions (SNNP and Oromia regions). The study participants were mothers who had under one year of infant during the data collection period.

### Study design

In the PMA study, a longitudinal panel survey design was used to generate data about maternal healthcare and infant care practices. Using the PMA-Ethiopia datasets, we have produced three manuscripts including the current one. The first manuscript entitled ‘Receiving quality antenatal care service increases the chance of maternal use of skilled birth attendants in Ethiopia’, and published in PLOSE ONE journal in December 2022 [23]. The second manuscript entitled ‘home-based optimal newborn care practice and associated factors among Mothers in Ethiopia: a community-based longitudinal panel survey’, and published in BMJ Open Journal in June 2023 [24]. In all manuscripts, the quantitative data generation process was almost the same.

### Study population

The source population was all mothers who had under-one year infants in the six regions during the PMA project period, from 2019 to 21. Whereas, the study population was all breastfeeding mothers who had under-one year infant and enrolled during pregnancy and interviewed at 6 weeks, 6 months and 1 year about infant feeding practice from selected enumeration areas in the six regions during the PMA project period, from 2019 to 21.

### Sample size and sampling procedure

The required sample size for the quantitative study was calculated using Stata vers.16 software considering the following assumptions: a 10% outcome difference between exposed and unexposed, a 16% baseline level of not received counseling on breastfeeding (key indicator) during pregnancy chosen from studies [17], a 5% level of significance, and 80% power. The required sample size was increased by design effect (Deff = 1.5) and incompleteness rate (10%). Hence, the estimated sample becomes 860.

The PMA Ethiopia panel survey employed a stratified two-stage cluster sampling design to select enumeration areas (clusters) and households from both urban and rural areas of the six regions. In the first stage, enumeration areas (EAs) were selected using probability proportional to size sampling technique from the national Central Statistical Agency (CSA) sampling frame, which contains information about the EA’s location, type of residence (urban, rural) and the estimated number of households in each EAs. In the second stage, 35 households per cluster were selected and all pregnant women aged 15–49 in the selected households were enrolled during pregnancy and interviewed after delivery. In this study, to increase the power of the study, all available 1,755 mothers of infants were included in the final analysis.

To address the qualitative objective, 14 participants were selected from urban and rural kebeles of an institution HDS or community attachment sites in two regions (SNNP and Oromia) using criterion purposive sampling technique. The final sample size in this study was determined based on information saturation among each group of the study participants (occupation). The eligible participants in each kebele were selected in collaboration with health workers in the areas.

### Quantitative data collection and measurement

In the PMA-Ethiopia panel survey, structured and standardized forms were used to enroll mothers during pregnancy and to interview them after delivery. Data were collected by using the Open Data Kit (ODK) system using tablet computers by trained field workers. Data on household location, housing and sanitation practices, economics and asset ownership, socio-demographic status and reproductive health details were collected during baseline census by trained field workers. Mothers and infants were followed and interviewed at 6 weeks, 6 months and 1 year of postnatal periods by trained community health extension workers. During follow-up visits data on maternal healthcare service utilization, and infant feeding and healthcare practices were collected by using a structured interviewer-administered questionnaire. The data collection was supervised by trained master’s degree graduates in the field of public health.

According to WHO Infant and young Child Feeding (IYCF) definitions: early initiation of breastfeeding is defined as the proportion of children born in the last 12 months who were put to breast within one hour of birth. Exclusive breastfeeding is defined as the proportion of infants 4–5 months of age who are fed exclusively with breast milk, and continued breastfeeding at 1 year is defined as the proportion of children 12–15 months of age who are fed breast milk. The introduction of complementary foods is defined as the proportion of infants 6–8 months of age who received solid, semi-solid or soft foods [14, 25].

In this study, completion of the continuum of age-appropriate infant feeding was generated and constructed from mothers’ responses on the time of initiation of breastfeeding after birth, exclusive breastfeeding until 6-months of age, and timely initiation of complementary feeding at six months of age, and dietary diversity (baby received food made from animal source and plant source foods). To determine and measure if a mother had initiated breastfeeding within an hour: mothers were asked the question of “How long after birth did you first put your infant to the breast?” To determine and measure if a mother had exclusively breastfed until 6 months: mothers were asked the question “For how long did you exclusively breastfeed?” To determine the start of complementary feeding: mothers were asked “When did you start infant complementary feeding?” Similarly, dietary diversity was measured by asking mothers whether their baby received food made from an animal sources (milk, meat, egg, and fish), and plant sources (grains/cereals, legumes (bean), veggies (root, dark green) and ripe fruits) in the previous day. Therefore, in this study infants are considered to receive diversified food if the infant had received food made from both animal and plant-based sources, as mentioned above. Finally, completion of the continuum of age-appropriate infant feeding was treated as a binary outcome and defined as “complete” if a mother practiced the four services, or “incomplete” if none or not practiced either of the four services.

The household wealth quintile was determined by giving scores based on the number and kinds of consumer goods they own, source of drinking water, type of toilet facilities and flooring materials. The score was derived using principal component analysis, and then households’ wealth index was divided into quintiles according to the wealth score as “lowest”, “lower”, “middle”, “higher” and “highest” [4].

### Qualitative data collection

First, an in-depth interview guide was prepared in English to explore mothers’ early initiation of breastfeeding after childbirth, exclusive breastfeeding in the first six months of age, the introduction of complementary foods such as solid, semi-solid or soft foods at six months of age and dietary diversity [14, 26]. Then, the interview guide was translated into Amharic and Affan Oromo language by fluent speakers of both languages. Then, back translated to English by another fluent speaker of the languages to check consistency. The data were collected using the interview guide by the principal investigator (Ph.D. student) and trained MPH holder as an assistant (Affan Oromo speaker). Each in-depth interviews were convened at the participant’s home or compound where there was unlikely to be interruption or excessive noise interference and convenient for participants. Interviews were commenced with introductions and clarifications about the purpose and procedures of the interview. Interviews were conducted in Amharic or Afan Oromo (as required) for 15 to 25 min. The interviewer introduced and guided the interview discussion (kept the interview focused and managed interview time). Audio recording of the in-depth interviews was done and field note taking and expansion were done on the same day after each interview.

In this study, credibility was maintained by diversifying the study participants in terms of area of residence, age and occupation. To maintain transferability efforts were made to obtain detailed responses using appropriate probes, and to ensure conformability care was taken by the researcher not to introduce the researcher’s prior experience in all data processes. To ensure dependability pre-tested interview guide was used and efforts were made to build trust among participants and other means.

### Quantitative data analysis

The quantitative data were formally requested and downloaded from the PMA Ethiopia website [27–30]. Then, data were cleaned and prepared for merging using Stata ver.16 software. The baseline and the follow-up visits datasets were merged using the study participant identity number (participant ID). The appropriate sample was restricted based on the design and inclusion criteria to address the study objective. Description of the study participants, unweighted and weighted (sampling weight) frequency, by sociodemographic characteristics was computed and presented using tables.

As we have binary outcome and clustered data, the generalized estimating equation (GEE) logistic regression model was fitted to account for the correlation of observations within an enumeration area (EA), considering EA as a clustering variable and controlling the effect of potential confounders. The association between each potential exposure and outcome variables was assessed. The odds ratio (OR) with the corresponding 95% confidence interval (CI) was computed to measure the association between exposure and outcome variables. Then, exposure variables that were statistically significant at *p* < 0.20 during the GEE binary logistic regression model were selected as a potential confounder variable for GEE multiple logistic regression. The GEE multiple logistic regression model was fitted to control the confounding effect of the variables. Area of residence (urban, rural), women’s place of delivery (home, health facility), number of ANC visits ( > = 4, < 4 visits), educational level (never attended, primary, secondary, vocational and higher), and wealth quintile (lowest, Lower, middle, higher, highest) were included in the final model. A *p* < 0.05 was used for the final interpretation of the statistically significant association between the exposure and outcome variables.

### Qualitative data analysis

Verbatim transcription of audio records was done within a few days by the principal investigator. The interview data (transcripts) were cleaned for errors through repeated reading. A content-driven theme approach (thematic approach) was used. Codes were created from the interview data itself, following an inductive coding approach. Then, codes are applied to the text. Characteristics are coded and reduced into core categories (themes) using Atlas.ti7 software and themes (context formulated) were described.

## Results

In this study, the quantitative and qualitative data were analyzed concurrently, and the findings are integrated in this section.

### Socio-demographic characteristics of quantitative study participants

In the quantitative study a total of 1,755 mothers of infants were included from six regions of Ethiopia. The majority of the study participants were from Oromia (44%), SNNP (22.5%), and Amhara (22.0%) regions. Over two-thirds of the study participant mothers (78.2%) were from the rural parts of the regions. Nearly 96% of the study participant mothers were married, and nearly 42% of them had never attended formal education, followed by 39.1% attending primary school (Table 1).

**Table 1:** Socio-demographic characteristics of mothers of infants in six regions of Ethiopia (*n* = 1,755), 2019-21

Socio-demographic characteristics	Weighted percent	Weighted *N*	Unweighted *N*
**Region**			
Oromia	44.2	776	443
SNNP	22.5	395	414
Amhara	22.0	388	324
Tigray	6.1	106	282
Addis Ababa	3.7	64	171
Afar	1.5	26	121
**Area of residence**			
Rural	78.2	1,373	1,066
Urban	21.8	382	689
**Household wealth index**			
Lowest quintile	20.9	366	291
Lower quintile	19.3	340	259
Middle quintile	20.3	356	277
Higher quintile	19.7	345	329
Highest quintile	19.8	348	599
**Women Age (in years)**			
15–19	9.0	158	133
20–24	23.7	416	424
25–29	31.2	547	580
30–34	19.5	343	350
>=35	16.6	291	268
**Marital status**			
Married/in union	95.8	1,682	1,657
Single	4.2	73	98
**Religion**			
Orthodox	37.7	659	806
Islam	34.9	610	511
Protestant	26.0	456	421
Other*	1.4	30	17
**Education level**			
Never attended	42.6	747	663
Primary	39.1	686	633
Secondary	11.2	197	260
Vocational & higher	7.1	125	199
**Baby gender**			
Male	51.5	904	895
Female	48.5	851	860

*Catholic, Wake-feta, Traditional

### Level of completion of the continuum of age-appropriate infant feeding practice

This study showed that 13.96% (95% CI: 12.4 to 15.6%) of mothers practiced a complete continuum of age-appropriate infant feeding. Over 8% of mothers did not practice any optimal feeding. Approximately 47% of mothers practiced optimal breastfeeding. Similarly, nearly one-fifth of mothers practiced optimal complementary feeding. (Table 2)

**Table 2:** level of completion of the continuum of age-appropriate infant feeding practices among breastfeeding mothers in Ethiopia, 2019-21

Variables	Frequency (*N* = 1755)	Percentage (%)
Practiced a complete continuum of age-appropriate infant feeding **(yes)**	245	13.96
Initiated breastfeeding within an hour after delivery **(yes)**	1135	64.67
Practiced exclusive breastfeeding (EBF) up to 6 months **(yes)**	1240	70.65
Introduced complementary food at 6 months of infant age **(yes)**	1,089	62.05
Practiced dietary diversity **(yes)**	549	31.28
Practiced optimal breastfeeding **(yes)**	820	46.72
Practiced optimal complementary feeding **(yes)**	355	20.23
Did not practice any of optimal feeding **(yes)**	147	8.37

### Barriers to the continuum of age-appropriate infant feeding practice

During the binary analysis of the quantitative data, mothers’ area of residence, educational level, household income level, and place of delivery were found to be statistically significantly associated with mothers’ completion of the continuum of age-appropriate infant feeding practice at a *p* < 0.20. However, after adjustment for the potential confounders, none of the selected variables were statistically significantly associated with mothers’ completion of the continuum of age-appropriate infant feeding practice at a *p* < 0.05 (Table 3).

**Fig. 1:**
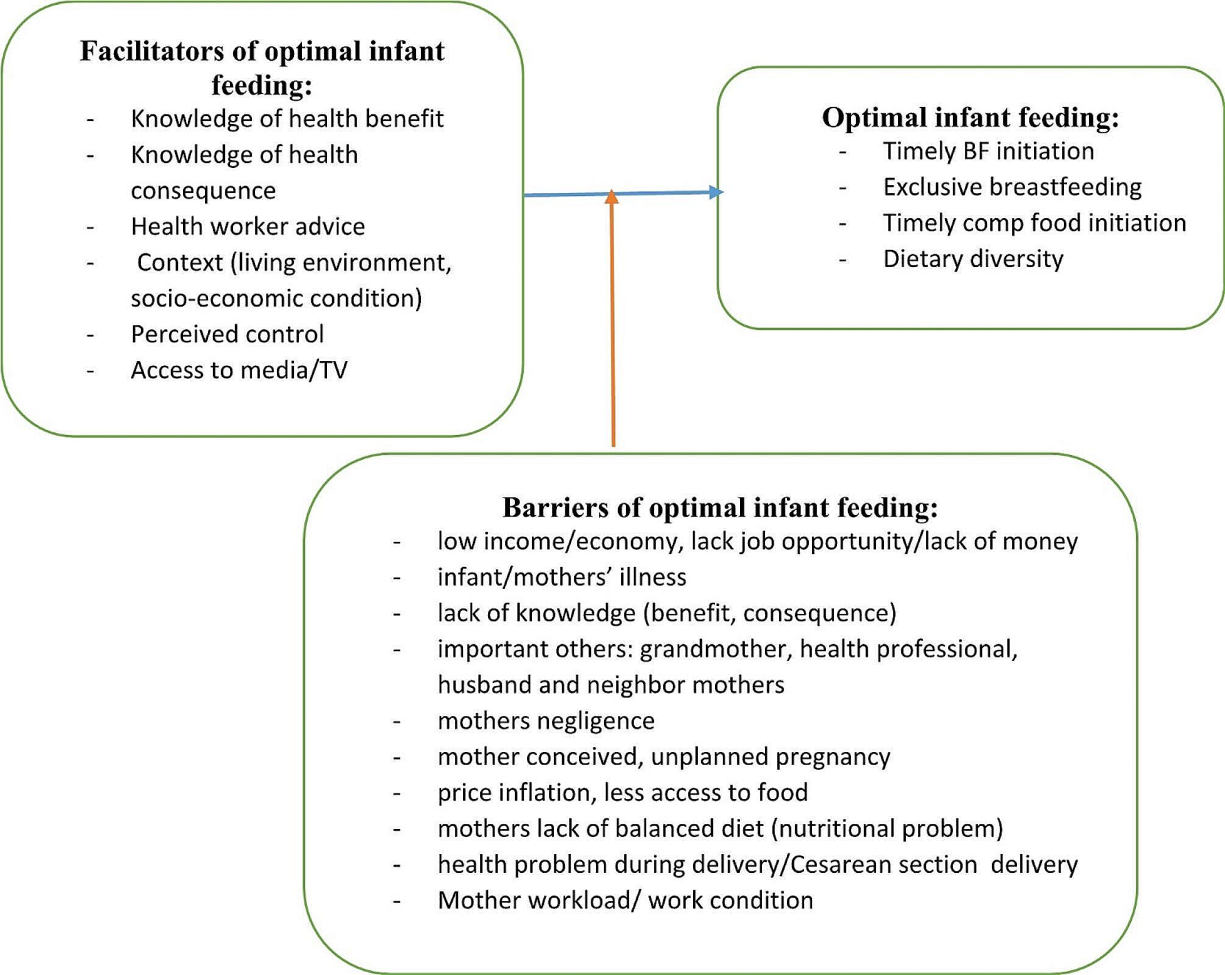
Facilitator and barriers of continuum of age-appropriate infant feeding practice

The qualitative findings showed that mothers optimal infant feeding practices were influenced by their low or limited source of income, their limited knowledge and attitude towards optimal feeding benefit and consequence, mother themselves and/or infant ill health status, important others (grandmother, husband, and health professional), price inflation, less access to food, and their workload or busy work conditions (Fig. 1).

The theory of planned behavior (TPB) was utilized as a framework to identify themes through the combination of quantitative and qualitative data. The results were then combined and explained in terms of the selected theme areas, which include perceived consequences, perceived social pressure, perceived behavioral control, and background factors (socio-demographic and knowledge factors).

### Background factors

The chance of completion of the continuum of age-appropriate infant feeding practice was 18% times lower among mothers of rural infants compared to mothers of urban infants (crude OR = 0.82, 95%CI = 0.59, 1.14). Similarly, the chance of completion of age-appropriate infant feeding practice was 31% times lower among mothers not attended formal education compared to mothers with vocational & higher education.

In the PMA survey, mothers’ knowledge of age-appropriate infant feeding practices was not measured, but this variable was measured in the qualitative interview. Many of the participant mothers correctly described the content and benefits of age-appropriate infant feeding practices.

“… *the initial breast milk (colostrum) is important for the infants and protects them from disease. It has benefits for the mother too: prevent breast distension (pain), helps to have normal weight, and it also increases mother-child psychological attachment.” (mother, age 27 years)*.

*“Breast milk is adequate until six months. It contains all the important things (nutrients) for the infant. After six month infants should get additional foods for their proper growth. Now I am feeding breast milk only. After six months, I have the plan to feed an additional variety of foods, like cerifam, carrot, potato, porridge and milk.” (Mother, 30 years)*.

In the qualitative interview, some mothers described that additional food mainly cow milk can be given to the infant at an earlier age, as breast milk is not enough or no problem with giving additional food (milk) before six months.

“*I don’t think breast milk is enough up to six months. I gave cow milk after diluting it with water at the age of three months because my breast did not produce enough milk, and the child might get hungry. The child should exercise bottle feeding at the age of three months. So far my child has not gotten any health problems, as a result of early additional food introduction*.” (Mother, 20 years age).

“*Up to six months infants should be fed breast milk only, but if you are an employee you can start additional food starting from 5 months to fulfill the energy requirements of the infant. I have got this advice from doctors (health professionals). Up to now, I am at home so that did not start any additional food, but after this month I will return to work, so I have to start additional food mainly cow milk as per the advice.”* (Mother, 25 years old).

### Perceived consequences

Similarly, in the PMA survey, the mother’s attitudes towards age-appropriate feeding practices not measured, but this variable was measured in the qualitative interview. Many of the participant mothers correctly described the negative consequences of inappropriate infant feeding practices.

“*up to six months a child should only feed breast milk. If a child is given additional food before six months, many problems will occur. The child becomes at risk of infection (disease). For example, I started bottle feeding at the age of two months because of the two fasting month. This led my child to diarrheal disease. She had diarrhea five times a day. She was seriously affected. Then, I stopped bottle feeding and breastfeeding up to six month. Early bottle feeding affects their health, especially if there is a hygiene problem. The bottle may be contaminated by flies, but mother breast milk is clean and safe.*” (Mother, 27 years age).

*“If a child is not appropriately fed, the child’s growth and mental development will be compromised, and the child will not have a bright mind. The child also lacks strength. The other thing, is the child will be easily susceptible to disease and cannot resist disease*.” (Mother, 30 years age).

*“If an infant is not properly fed, their growth will be delayed, lack strength and be unable to walk. If they do not get a balanced diet, like, eggs, fish, vegetables, milk and others, their growth will be compromised. They become malnourished (wasted) and will not have bright minds. … They may develop anemia and become at risk of disease.”* (Mother, 24 years age).

### Perceived social pressure

Compared to mothers who delivered at home, the chance of the completion of continuum of age-appropriate infant feeding practice was 26% times higher among mothers who delivered at a health facility (Crude OR = 1.26, 95%CI = 0.94,1.68).

In the qualitative interview, some mothers described that important others including health professionals, husbands and grandmothers can positively or negatively influence child-feeding practices.

“*We practice optimal feeding as per the health professional advice during pregnancy visits and after delivery. They tell us how to care for and feed our children. They tell us to expose the baby to morning sunlight for 10 minutes after delivery, and for how long the infant breastfeeds. Based on what we have heard we practice at home.”* (Mother, 27 years age).

“ *A neighbor nurse professional (female) told me that it is possible (no associated problem) to give cow milk after diluting with water from the age of three months. The nurse herself is giving cow milk after diluting it with water. She has been given cow milk because she works at a health office (Woreda health office) so that not convenient for her to breastfeed; however, her breast not produce enough milk, like mine. To fulfill the child’s energy need, I should feed cow milk because we do not want our child to get hungry. … In addition to breastfeeding, I start other foods feeding after six months of infant age. Up to one year, I feed the infant mainly rice and Endomi (processed fast food). Then, after one year I feed him what we family members eat.”* (Mother, 20 years age).

“*Mothers (Grandmothers) have a great role in child feeding practice. They tell you to give water and herbal medicine to prevent abdominal cramps especially if the baby is crying. For example, my mother gave water to my baby at an earlier age and she developed diarrhea. Then, I took her to a health facility and doctors (health professionals) identified Giardia and treated her.”* (Mother, 27 years age).

### Perceived behavioral control

The chance of completion of age-appropriate infant feeding practice was 37% times lower among mothers with the lowest household income compared to mothers with the highest household income.

The finding from the qualitative study showed that mothers may not practice optimal infant feeding due to inconvenient work conditions and low income (lack of job opportunity/lack of money), price inflation and less access to food.

*“optimal complementary feeding depends on what you have. If you have access, it is good to feed them different varieties, like, eggs, meat, vegetables…, but if you don’t have access, you can do nothing what you can do is give what you have already.”* (Mother, 24 years age).

*“If you are expecting expense from a husband you may not feed properly as he may give a small amount of expense (money). Our (mothers) being busy with activities may influence not to feeding properly. She may leave home early in the morning and return at night from work (employed or unemployed), so the child will not get breast milk or not be properly fed.”* (Mother, 20 years age).

**Table 3:** *GEE multivariable logistic regression analysis for* continuum of age-appropriate infant feeding practice with independent variables, 2019-21

Variables	Age-appropriate infant feeding practice(*n* = 1,755)		Crude OR (95% CI)	Adjusted OR(95% CI)
	Yes (%)	No (%)		
**Area of residence**				
Rural	136(12.8)	930(87.2)	0.82(0.59,1.14)*	0.95(0.56,1.59)
Urban	109(15.8%)	580(84.2%)	1.00	1.00
**Educational level**				
Never attended	67(10.1)	596(89.9)	0.69(0.45,1.05)*	0.75(0.46,1.19)
Primary	107(16.9)	526(83.1)	1.01(0.69,1.48)	1.05(0.70,1.58)
Secondary	40(15.4)	220(84.6)	0.97(0.63,1.49)	0.99(0.64,1.51)
Vocational & Higher	31 (15.6)	168(84.4)	1.00	1.00
**HH Wealth index**				
Lowest quintile	28(9.6)	263(90.4)	0.63(0.40,1.02)*	0.78(0.41,1.51)
Lower quintile	35(13.5)	224(86.5)	0.91(0.61,1.36)	1.08(0.60,1.92)
Middle quintile	41(14.8)	236(85.2)	0.88(0.59,1.30)	1.03(0.58,1.82)
Higher quintile	46(13.9)	283(86.0)	0.83(0.57,1.20)	0.95(0.59,1.51)
Highest quintile	95(15.9)	504(84.1)	1.00	1.00
**Women age (in years)**				
15–19	18(13.5)	115(86.5)	1.04(0.62,1.73)	--
20–24	65(15.3)	359(84.7)	1.17(0.80,1.70)	--
25–29	78(13.4)	502(86.6)	0.98(0.68,1.42)	--
30–34	47(13.4)	303(86.6)	1.04(0.70,1.54)	--
>=35	37(13.8)	231(86.2)	1.00	--
**Place of delivery**				
Health facility	165(15.2)	917(84.8)	1.26(0.94,1.68)*	1.14(0.82,1.57)
Home	80(11.9)	593(88.1)	1.00	1.00
**Parity**				
<5 live births	197(13.7)	1244(86.3)	0.91(0.67,1.21)	--
>=5 livebirths	48(15.2)	267(84.8)	1.00	--
**Number of ANC visits**				
>=4 ANC visits	94(14.6)	550(85.4)	1.01(0.76,1.32)	0.89(0.67,1.20)
< 4 ANC visits	151(13.6)	960(86.4)	1.00	1.00
**Visited/seen HEW during pregnancy**				
Yes	69(13.7)	435(86.3)	0.86(0.63,1.16)	--
No	176(14.1)	1,075(85.9)	1.00	--
**Planned to feed other than breast milk in the first 6 months**				
No	154(14.3)	926(85.7)	1.08(0.80,1.46)	--
Yes	91(13.5)	584(86.5)	1.00	--

*Statistical significance at *p*-value < 0.20

## Discussion

The quantitative finding showed that only approximately 14% of mothers started and continued age-appropriate infant feeding practices in Ethiopia. Based on both quantitative and qualitative data, factors that influenced a mother’s continuum of age-appropriate infant feeding practice were identified, integrated, and described under the identified thematic areas. The thematic areas are background factors, perceived consequences, perceived social pressure, and perceived behavioral control. However, none of the background (socio-demographic) factors were statistically significantly associated with the outcome variable.

The World Health Organization recommended that mothers initiate breastfeeding within an hour after birth, exclusively breastfeed up to 6 months, continue breastfeeding for 1 and more years, introduce semi-solid or soft foods at 6 months, prepare minimum dietary diversity (≥ 4 food groups), and give minimum meal frequency (≥ 3 meals in a day) to promote infant and child healthy growth [31].

The current study showed that less than 14% of mothers started and continued optimal age-appropriate infant feeding practices in Ethiopia. This shows that nearly nine in 10 mothers did not start and continue age-appropriate infant feeding practices as per the recommendations. Similarly, some in-depth interview mothers described that only breast milk feeding until six months is not enough to fulfill the energy requirements of the baby, and optimal complementary feeding practice mainly depends on the availability and access to diverse foods items. Therefore, the health offices and concerned local stakeholders should collaborate to increase the continuum of age-appropriate infant feeding practice in Ethiopia by strengthening families’ education on the health benefits of optimal infant feeding activities and designing and promoting strategies to improve household income or household access to diverse food.

Although no previous study estimated the level of the complete continuum of age-appropriate infant feeding practices by mothers, some studies reported the level of exclusive breast feeding and timely initiation of complementary feeding practices by mothers separately, which ranged from 10 to 88% and 34 to 83%, respectively [15–17, 19, 29–42]. Similarly, studies conducted in Ethiopia and in urban slums in India reported that proportions of mothers who initiated breastfeeding within an hour after delivery ranged from 23 to 63% [33, 36, 37]. Also, a study conducted in Ethiopia reported that about 24% of children had good dietary diversity, and about 33% of children were fed with an appropriate meal frequency [43].

The current study showed that none of the assessed background (socio-demographic) factors were statistically significantly associated with mothers’ complete continuum of age-appropriate infant feeding practice; however, over three-quarters of mothers practiced suboptimal infant feeding in different categories of socio-demographic variables, including area of residence, household income, educational status, and place of delivery. The possible explanation could be that the current study may be underpowered to identify potential factors since only 245 mothers had a continuum of feeding practices. When we stratify these 245 into the different categories of the various variables included in the final model, we may not get a significant result. The other possible explanation could be use of secondary data, which may miss data on some potential confounder variables, including knowledge and attitude towards optimal infant feeding practice.

In this study, the effect of mothers’ knowledge and attitude on the completion of a continuum of age-appropriate infant feeding practice was not assessed due to the data limitations of the PMA survey. However, some in-depth interview mothers described that optimal infant feeding has several benefits, like helping children to have proper growth and mental development (a bright mind), getting strength and resisting diseases. Similarly, studies conducted in Ethiopia and Kenya reported that optimal exclusive breastfeeding and complementary feeding practices were higher among mothers who were knowledgeable about the timing and benefits of optimal child feeding [19, 21, 33, 44]. Also, a study conducted in rural Kenya reported that the chance of a mother’s early cessation of exclusive breastfeeding was lower among mothers who had knowledge of breastfeeding and had positive beliefs about the impact of exclusive breastfeeding on children and their perception of impact of exclusive breastfeeding on mother [44].

The current study also showed that higher proportions of mothers who gave birth at health facilities started and continued age-appropriate infant feeding practices compared to mothers who gave birth at home. The possible explanation could be that mothers who gave birth at a health facility might receive counseling on optimal child feeding practices during pregnancy visits and delivery by health professionals. In some in-depth interviews, mothers described that important others, including health professionals, grandmothers, and husbands, have great influence on practicing age-appropriate infant feeding. Studies conducted in rural Kenya and India reported that the chance of exclusive breastfeeding was higher among mothers who had a positive perception of the acceptability of exclusive breastfeeding by important others and had a supportive norm [44, 45].

The current study showed that nearly only 15% of mothers in all income categories practiced optimal age-appropriate infant feeding. This shows that mothers with no economic problems still practiced suboptimal feeding. The possible explanation could be mothers with higher income may have limited knowledge and attitude towards optimal infant feeding practices; however, economic problems may be a significant major barrier for mothers with lower household income. Some of the in-depth interview participants described that low income and less access to food can significantly influence mothers’ age-appropriate infant feeding practices. This finding was in line with studies conducted in Ethiopia and India, which reported that low socioeconomic status was a major barrier to practicing age-appropriate infant feeding [17, 18, 45, 46].

Using a sequential explanatory mixed method study has many strengths. The unequal selection probability of mothers of infants and the clustering nature of the data by enumeration area were accounted for in the quantitative analysis, so the findings can be reliable and used to make generalizations. As a limitation, this study might be at risk of Hawthorne effect bias, as the study mothers might change in their practice of infant feeding because they knew their baby, including themselves, was being studied. This limitation might not be a problem in this study, as the misclassifications were more likely to be non-deferential in the enumeration areas. In addition, in this analysis, the effect of mothers’ knowledge and attitude towards infant feeding benefits and consequences on the outcome was not assessed due to data limitations in the PMA survey, but the effect of these variables on the outcome was explored qualitatively. Therefore, the qualitative study results would make the quantitative study results interpretation complete and reliable. Even though there was time lag between the quantitative and qualitative studies, there might not be a total change in barriers to optimal feeding practices after the quantitative study.

## Conclusion

From the findings of the current study, we can conclude that the level of complete continuum of age-appropriate infant feeding practice is low among breastfeeding mothers in Ethiopia. Mothers’ complete continuum of age-appropriate infant feeding practice is affected by their knowledge and attitude towards optimal child feeding, economic status, and health professionals, and family members. Therefore, public health planners and health professionals in the system, including health extension workers, should instruct and encourage mothers to practice age-appropriate feeding by explaining the benefits of optimal feeding and the consequences of suboptimal feeding. Health extension workers, together with concerned local stakeholders, should advise and encourage husbands and grandmothers to support mothers in practicing age-appropriate infant feeding. Decision makers and concerned stakeholders should also give priority attention to mothers of infants with low household income to improve their source of income.

## Data Availability

The quantitative datasets used and analyzed in the current study are available upon reasonable request from the Performance Monitoring for Action website via https://datalab.pmadata.org/datasets. Whereas, the in-depth interview data are available from corresponding author on reasonable request.

## Abbreviations

PMA: Performance Monitoring for Action
SNNP: Southern Nations Nationality People
QUAN: Quantitative
QUAL: Qualitative
HDS: Health and Demographic sites
EAs: Enumeration Areas
CSA: Central Statistical Agency
ODK: Open Data Kit system
WHO: World Health Organization
IYCF: Infant and Young Child Feeding
MPH: Masters of Public Health
OR: Odds Ratio
GEE: Generalized Estimating Equation
ANC: Antenatal Care
TPB: Theory of Planned Behavior
CI: Confidence Interval
HEWs: Health Extension Workers
CS: Caesarian Section delivery
